# A Giant Mesenteric Cyst in a Prepubertal Girl Mimicking an Ovarian Mass: A Case Report

**DOI:** 10.7759/cureus.91436

**Published:** 2025-09-01

**Authors:** Lakshay Singla, Prem Chand, Paramjit Kahlon

**Affiliations:** 1 General Surgery, Government Medical College Patiala, Patiala, IND

**Keywords:** abdominal lump, abdominal pain in females, diagnostic dilemma, ovarian mass differential diagnosis, primary mesenteric cyst

## Abstract

Mesenteric cysts are rare benign intra-abdominal lesions in children, often presenting with nonspecific symptoms and posing diagnostic challenges. Their clinical and radiological resemblance to ovarian or paraovarian pathologies may lead to misdiagnosis, especially in female patients. We report the case of an eight-year-old premenarchal girl who presented with chronic abdominal pain and a palpable abdominopelvic mass. Imaging studies revealed a large multiloculated cystic lesion suggestive of a paraovarian cyst or serous cystadenoma. An exploratory laparotomy uncovered a giant mesenteric cyst measuring approximately 12 cm, adherent to the jejunal wall. Due to dense adhesions, complete cyst enucleation was not feasible, and segmental bowel resection with anastomosis was performed. Histopathology confirmed a benign mesenteric cyst. The patient had an uneventful postoperative recovery and remained asymptomatic during follow-up. This case highlights the diagnostic dilemma posed by mesenteric cysts in paediatric patients and emphasizes the importance of surgical exploration for definitive diagnosis and treatment. Early recognition and timely management result in excellent outcomes.

## Introduction

Mesenteric cysts are uncommon benign intra-abdominal lesions, with an estimated incidence of one in 100,000 to 250,000 hospital admissions [1]. These lesions are especially rare in the pediatric population [2], accounting for a small fraction of abdominal masses in children. Most mesenteric cysts are found in the mesentery of the small intestine, particularly the ileum [3], and they exhibit a wide range of presentations depending on their size, location, and contents.

Clinically, patients may present with vague abdominal pain, a palpable mass, or even an acute abdomen due to complications [4] such as volvulus, hemorrhage, rupture, or intestinal obstruction. In prepubertal female patients, mesenteric cysts can be radiologically mistaken for ovarian or paraovarian cysts [5], making preoperative diagnosis particularly challenging.

Imaging modalities such as ultrasonography and CT can suggest the cystic nature of the lesion [6] but often fail to accurately determine its origin. Definitive diagnosis is usually made intraoperatively [7] and confirmed on histopathology. Complete surgical excision is the treatment of choice [8] to prevent recurrence or complications. Segmental bowel resection may be necessary [9] when the cyst is densely adherent to the bowel or involves mesenteric vessels.

We present a case of a giant mesenteric cyst in an eight-year-old girl that radiologically mimicked a paraovarian lesion and required segmental jejunal resection for complete removal. This case underscores the importance of maintaining a broad differential diagnosis for cystic abdominal masses in prepubertal girls.

## Case presentation

An eight-year-old premenarchal girl presented to the surgical outpatient department of Government Medical College and Rajindra Hospital, Patiala, in April 2016 with complaints of gradually progressive abdominal pain for three months. The pain was dull, nonradiating, and not associated with vomiting, fever, or altered bowel habits. There was no history of jaundice, weight loss, bleeding per rectum, or exposure to pets.

On physical examination, a palpable, nontender mass was noted in the periumbilical region. The lump had a smooth surface, positive fluctuation, and moved with respiration. Its margins could not be clearly delineated.

Investigations

**Figure 1:**
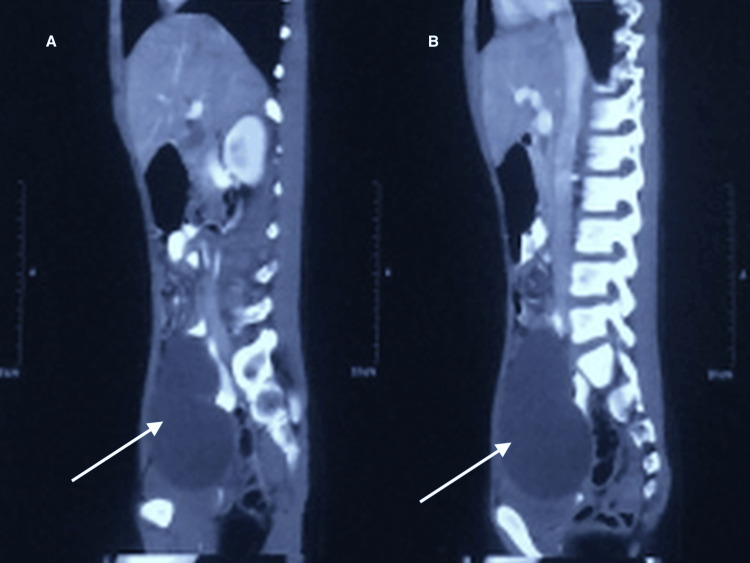
CECT abdomen and pelvis CECT: contrast-enhanced computed tomography CECT scan of the abdomen showing a large, multiloculated, well-marginated cystic lesion in the abdominopelvic region, initially suspected to be of paraovarian origin. Panels A-B showing saggital sections depicting large cystic lesion involving both abdomen and pelvis

Ultrasonography (image unavailable) and contrast-enhanced computed tomography (CECT) (Figure 1) of the abdomen revealed a well-defined, smooth-marginated, multiloculated cystic lesion measuring approximately 5.2 × 11.7 × 10.6 cm in the abdominopelvic region. The lesion was closely abutting the anterosuperior aspect of both ovaries, suggesting a possible paraovarian cyst or serous cystadenoma. Unfortunately, additional axial CT/MRI cuts, including the uterus and adnexal organs, were not available in our records; this limitation itself contributed to the initial diagnostic dilemma, as the lesion closely mimicked an ovarian origin. Baseline hematological and biochemical investigations were within normal limits. Hydatid serology (enzyme-linked immunosorbent assay (ELISA)) and tumor marker CA-125 were negative.

Surgical procedure

**Figure 2:**
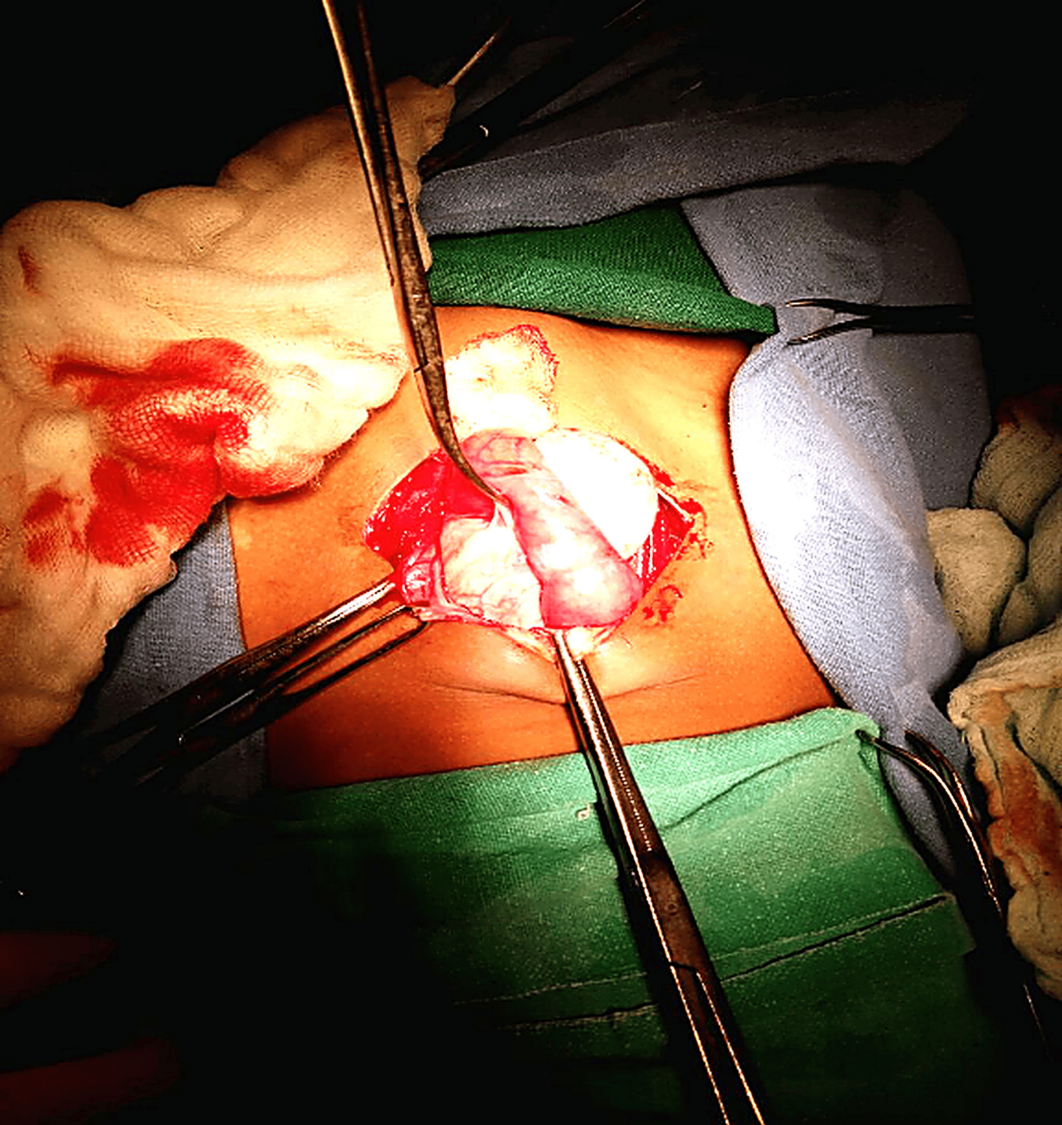
Intraoperative image following exploratory laparotomy Intraoperative view showing a large mesenteric cyst arising from the jejunal mesentery and densely adherent to the bowel wall

The patient underwent exploratory laparotomy with a lower midline vertical incision under general anesthesia. Intraoperatively, a large mesenteric cyst measuring approximately 5 × 12 × 11 cm was identified (Figure 2), arising from the jejunal mesentery and adherent to the wall of the jejunum. The bowel was systematically examined from the duodenum to the terminal ileum, and no additional pathology was identified. Both ovaries, fallopian tubes, and the uterus were found to be normal with no associated pathology.

**Figure 3:**
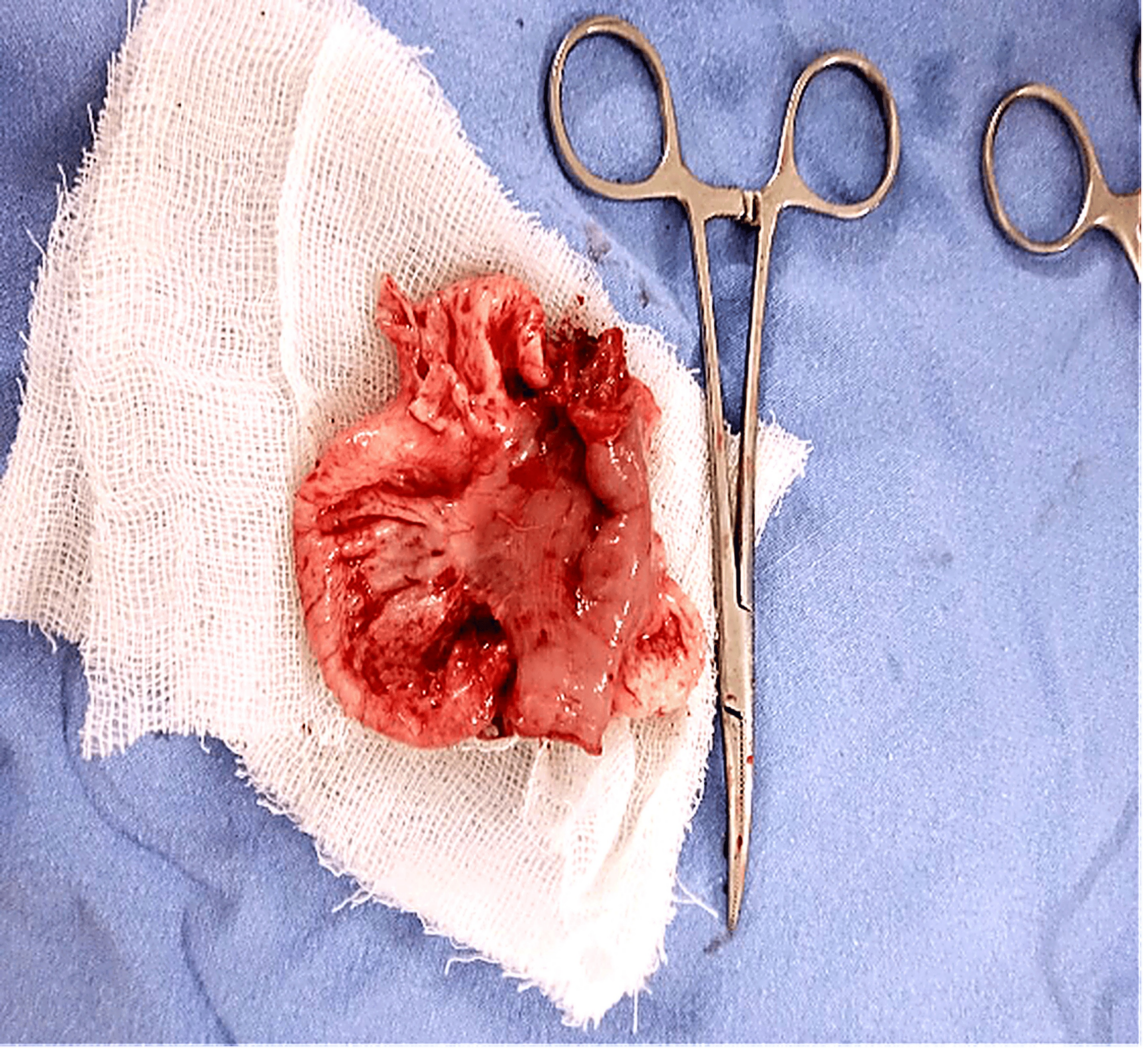
Excised specimen Excised specimen showing segment of jejunum with the attached mesenteric cyst

**Figure 4:**
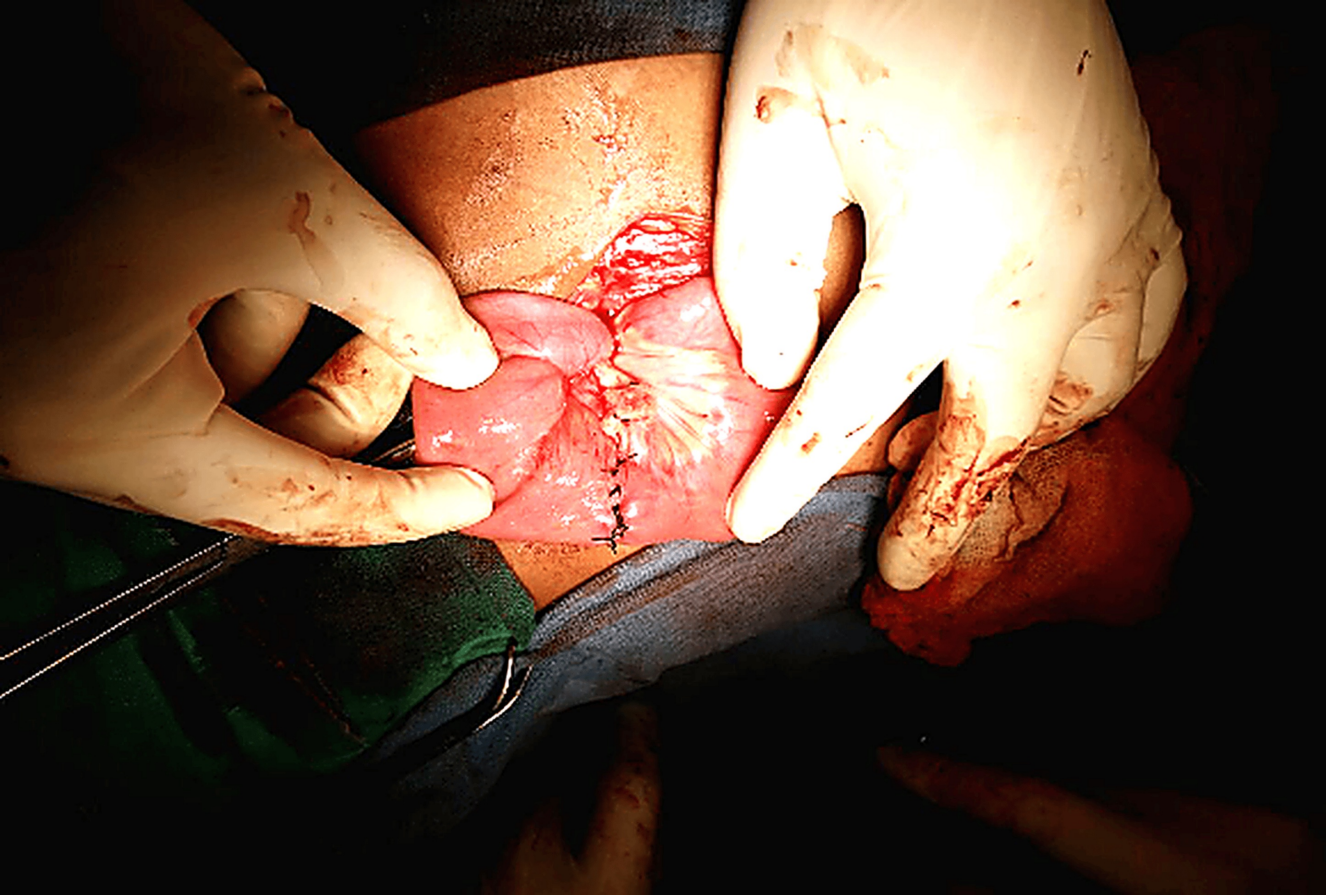
Intraoperative image after excision of the bowel and cyst Completed jejuno-jejunal anastomosis after resection of the involved segment

The cyst was aspirated, yielding approximately 600 mL of clear, light-yellow fluid, which was sent for cytological and adenosine deaminase (ADA) analysis. Due to dense adhesions with the jejunal wall, enucleation of the cyst was not feasible. Hence, the involved bowel segment was resected (Figure 3), and a primary jejuno-jejunal anastomosis (Figure 4) was performed. An abdominal drain was placed, and the abdomen was closed in layers.

Postoperative course

A nasogastric tube was inserted intraoperatively and removed on postoperative day 1 once bowel sounds returned. The patient was started on oral liquids the same day and advanced to solid food by postoperative day 2, which she tolerated well. The patient had an uneventful postoperative recovery. Drain output was serosanguinous and minimal, ceasing by postoperative day 3. The drain was removed on day 5, and the patient was discharged in stable condition. The patient was followed up for 12 months and remained healthy, with no recurrence or complications.

Histopathological findings

**Figure 5:**
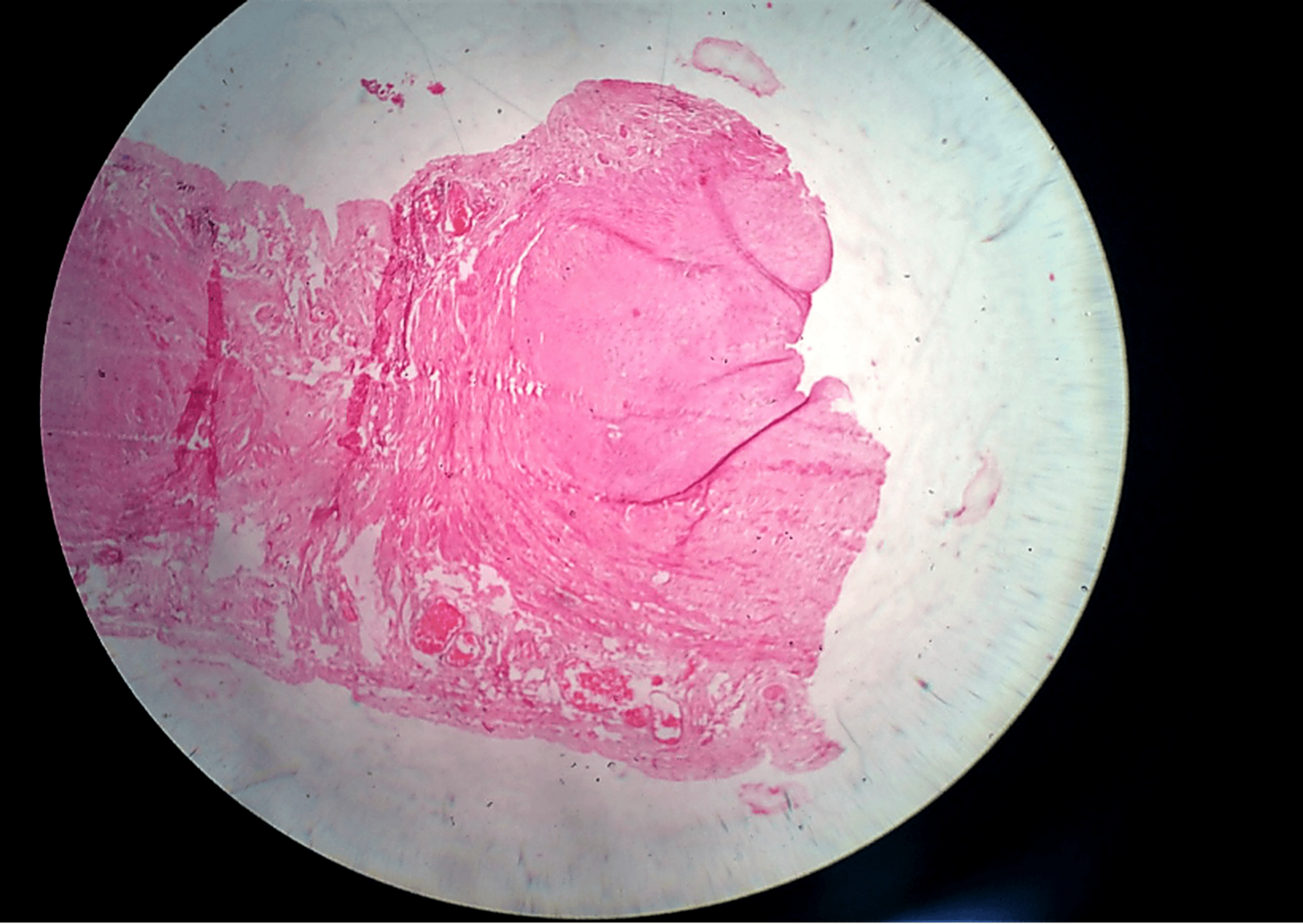
Histopathological Image H&E stain: hematoxylin and eosin stain Histopathological image showing fibrous cyst wall with chronic inflammation and myxoid degeneration (H&E stain, 100×)

Histological examination of the resected specimen revealed fibrous tissue with myxoid changes, chronic inflammatory infiltrate, focal haemorrhage, and congested blood vessels. Sections of the cyst wall showed intestinal tissue (Figure 5). Cytology of the aspirated fluid showed chronic inflammatory effusion with mild lymphocytosis. ADA testing was negative.

## Discussion

Mesenteric cysts are rare intra-abdominal entities in pediatric patients and can pose significant diagnostic challenges. While their etiology remains uncertain, prevailing theories suggest developmental anomalies of lymphatic tissue [10], traumatic disruptions, or obstruction of lymphatic channels. The most widely accepted explanation is benign proliferation of ectopic lymphatics [11] within the mesentery that lack communication with the central lymphatic system.

Approximately one-third of mesenteric cysts occur in children [12] under the age of 15 years. Presentations vary and are often nonspecific. In children, these cysts may manifest with abdominal pain [13], distension, nausea, or a palpable mass. Due to their rarity and nonspecific features, they are frequently misdiagnosed as other intra-abdominal or pelvic pathologies, as in our case, where the radiological appearance mimicked a paraovarian cyst.

Ultrasonography is typically the first-line imaging modality, but it may not always localize the cyst origin accurately. CT or MRI may help delineate the lesion further [14], especially when differentiating from ovarian or hydatid cysts. In our case, despite cross-sectional imaging, the lesion’s proximity to the ovaries led to an erroneous initial diagnosis of a paraovarian mass.

The treatment of choice is complete surgical excision. Enucleation is preferred when feasible, as incomplete excision can lead to recurrence [15] or complications such as infection, rupture, or hemorrhage. However, when the cyst is densely adherent to adjacent bowel or mesenteric vessels, as in our patient, segmental bowel resection [16] with primary anastomosis becomes necessary. Minimally invasive approaches have been successfully applied in selected pediatric cases [17], but large or adherent cysts often require open surgery.

Histopathology confirms the diagnosis and rules out rare but reported cases of malignancy [18], which occur in less than 3% of cases. Our case demonstrated typical features of a benign mesenteric cyst with chronic inflammation and myxoid degeneration.

Early surgical intervention leads to excellent outcomes, and recurrence is rare [19] following complete resection. Our patient recovered uneventfully and remained asymptomatic at follow-up.

## Conclusions

Mesenteric cysts, though rare in children, should be considered in the differential diagnosis of abdominopelvic masses, especially in prepubertal girls and when imaging mimics an ovarian origin. Their nonspecific clinical and radiological features can mimic ovarian or paraovarian lesions, leading to diagnostic delays. Definitive diagnosis is often established intraoperatively. Complete surgical excision remains the cornerstone of treatment, and bowel resection may be required in cases with dense adhesions. Early intervention ensures favorable outcomes and minimizes the risk of recurrence.
